# Association between Mastery Motivation, Executive Function, and Daily Participation of Young Children with and without Global Developmental Delays

**DOI:** 10.3390/children11020220

**Published:** 2024-02-09

**Authors:** Chien-Lin Lin, Hung-Yu Lin, Pei-Jung Wang

**Affiliations:** 1 Department of Physical Medicine and Rehabilitation, China Medical University Hospital, Taichung 404327, Taiwan; dr5699@yahoo.com.tw; 2 Department of Occupational Therapy, Asia University, Taichung 413305, Taiwan; otrlin@gmail.com; 3 Department of Physical Therapy, Asia University, Taichung 413305, Taiwan

**Keywords:** participation, executive function, motivation, children, developmental delay

## Abstract

This study aimed to examine the group differences in participation level between children with and without global delays and to explore the associations between mastery motivation, executive function, and participation in young children with and without global developmental delays (GDD). Methods: we recruited 26 children with GDD aged 2 to 5 years and 26 children with sex- and mental age-matched developing typically (TD). The participants were assessed child development using the standardized developmental test, and their mothers were asked to fill in questionnaires, including the revised Dimension of Mastery Questionnaire (DMQ 18) with preschool version to assess mastery motivation, the Behavior Rating Inventory of Executive Function with preschool version (BRIEF-P) to assess executive function, and the Young Children’s Participation and Environment Measure (YC-PEM) used to obtain participation levels. Results and conclusions: young children with GDD showed significantly lower participation levels at home, daycare, and community than TD group. We found that for young children, child mastery pleasure, health condition, and total persistence were significant predictors of child participation. Therefore, coaching parents to observe and facilitate their children’s motivation and executive function, as well as child developmental abilities, is important in order to enhance children’s participation in daily activities.

## 1. Introduction

The primary goal of early childhood intervention services for children with developmental disabilities is to enhance their participation in daily activities [1,2,3]. This goal is recognized as crucial for children’s overall development, health, and quality of life [3,4]. Participation is characterized as active involvement in various life situations [1]. The Participation concept has two essential constructs, including attendance and level of involvement. Attendance is defined as “being there”, and it is commonly measured by diversity and frequency. The level of involvement is defined as the experiences of participation while attending and is measured by the level of engagement and affect [3]. Previous studies have demonstrated that children with disabilities at school age exhibited lower participation attendance in social and recreation activities at home, school, and community settings than age- and sex-matched children with typical development [5,6,7,8]. What’s worse, low children’s participation has contributed to obesity and social isolation in children with disability [9,10]. Thus, it is crucial to examine associated factors influencing participation patterns in toddlers and preschoolers.

Children experiencing global developmental delay (GDD) exhibit obvious delays in reaching developmental milestones in two or more of the following areas: cognition, gross/fine motor skills, language, social interactions, and self-help activities [11]. The estimated prevalence of GDD in pediatric practice is approximately 1 to 3% [12]. Significant delay is defined as children’s performance falling at least two standard deviations below the mean on standardized norm-referenced developmental tests [11,12]. Previous research indicates that young children with GDD participated less frequently and were less engaged in preschool and community settings [6,13,14]. Furthermore, children with GDD have significant restrictions in school participation that are associated with child health conditions, child motivation, and executive function [9,15,16].

According to The International Classification of Functioning, Disability, and Health (ICF) framework, child participation is influenced by the child’s health condition (such as developmental status), child biosocial function (i.e., mastery motivation and executive function), and contextual (personal and environmental) factors [1,2,17,18,19]. Executive function is defined as a high-level cognitive process required for complex goal-directed behavior, and it contains three core components, including working memory, which refers to the ability to retain and update information over a brief period. Inhibitory control involves mastering and filtering thoughts and impulses to resist temptations or distractions, and cognitive flexibility is the ability to swiftly adapt and adjust to changing demands [20,21,22]. The advancement of executive functions and child cognitive abilities plays a crucial role, as the processes associated with these concepts frequently impact an individual’s effectiveness in executing complex tasks such as participating in daily activities [20,21]. Another child self-organization component is mastery motivation. Mastery motivation is characterized as a psychological drive that motivates individuals to persistently strive to master tasks, especially those that pose at least a moderate level of challenge for them personally, even in the face of initial unsuccessful attempts [23]. There are at least three domains of mastery motivation: cognitive (attempts to solve tasks or problems), social (attempts to master interpersonal relationships with adults and with peers), and gross motor (attempts to master physical skills) [24]. Within each domain, task-directed persistence (a child’s focused and persistent attempt to solve problems or master tasks) and affection (such as mastery pleasure and negative reactions to challenges) are used as behavioral indicators of mastery motivation [25]. Child EFs and mastery motivation are core components of child self-regulation processes, and they are theoretically associated with a child’s daily participation based on the ICF.

In terms of empirical studies for children with disabilities, several studies have found that child health conditions (such as the severity of developmental delay) and child self-organization process (such as executive function and mastery motivation) contribute to children’s participation in daily activities [9,15,26,27,28,29,30]. Some studies have indicated that child developmental abilities or severity of delay are significantly associated with child daily participation attendance [9,15,19,26,27] in children with disabilities. The positive association between mastery motivation and participation attendance in children with delays was found in two studies [19,28]. Two previous studies have shown that child mastery motivation and executive function are positively associated with school participation in school-age children with autism [28] or adolescents and young adults with physical disabilities [29]. Specifically, one study has found that child inhibitory control is significantly associated with participation attendance in preschoolers with disabilities [30]. For children with typical development, one study has reported that children with high mastery motivation showed better participation attendance for school-age children [31]. The positive association between executive function and daily participation in children of elementary school age was found in a previous study [32,33]. Furthermore, a longitudinal study has indicated that child mastery motivation predicted later school readiness through executive function in preschool children with typical development [34]. Although several studies have found a positive association among child developmental abilities, child mastery motivation or executive function, and one participation component (such as participation attendance) in children with and without delays, most of the studies focused on children of school age or even older age. Very few studies further examined the above-associated factors for predicting two participation components in young children with global delays. However, as far as our knowledge extends, there is limited research exploring the associated factors contributing to the two components of participation (attendance and involvement) in young children, both with and without delays.

Therefore, three aims of this study were the following: (1) to examine the differences in participation level between young children with global developmental delay (GDD) and those with typical development (TD) who were sex- and mental age-matched; (2) to explore the participation pattern in young children with GDD; (3) to examine the relationships between developmental abilities, mastery motivation, executive function, and participation in GDD and TD group. We hypothesize that: (1) young children with GDD would show lower levels of participation frequency and involvement in three different settings than their TD peers; (2) highest participation attendance and involvement at home setting would be found in young children with GDD when compared to other two settings; (3) a notable correlation would be expected between mastery motivation and daily participation at three settings in GDD group and TD groups; (4) there would be significant correlation between executive function and daily participation at three settings in GDD and TD groups.

## 2. Materials and Methods

### 2.1. Study Design

This study design was case-control conducted at a university laboratory in Taiwan.

### 2.2. Participants

Seventy-one participants provided consent for our study. We recruited young children with Global Developmental Delay (GDD) from clinics or daycare centers in the greater Taichung area. Inclusion criteria for caregivers and their children with GDD were as follows: (1) child aged between 24 to 60 months; (2) child diagnosed by a doctor with GDD, showing developmental delay in at least two domains; (3) receiving at least more than 4 h of daily care from the primary caregiver; and (4) the caregiver having a minimum educational level of junior high school. Exclusion criteria comprised: (1) autism spectrum disorder or attention deficit hyperactivity disorder; (2) progressive diseases like neuromuscular dystrophy or brain tumor; (3) children with unstable medical conditions (e.g., epilepsy), severe heart disease (e.g., Tetralogy of Fallot), frequent hospitalization, or those who underwent surgery over the last six months; and (4) impaired visual or auditory capacities, despite the use of supportive devices. For each child with GDD, we recruited a typically developing (TD) child matched in sex and mental age through advertisements or daycare centers.

For each child diagnosed with GDD, we calculated the mental age and then selected a typically developing (TD) child of the same sex, ensuring that the TD child’s chronological age was within a 2-month range of the GDD child’s mental age, using the standardized developmental test, Comprehensive Developmental Inventory for Infants and Toddlers (CDIIT). The inclusion criteria for TD children were as follows: (1) achieving a developmental quotient of 85 or higher on the CDIIT, (2) receiving at least more than 4 h of daily care from the primary caregiver, and (3) the caregiver possessing an educational level of at least junior high school.

Nine children with GDD did not meet our inclusion criteria, and two mothers opted not to proceed with the laboratory observation. As a result, data collection was completed with 60 dyads. Among these 60 dyads, the data for 8 dyads was excluded for further data analyses: 5 children exhibited delays in a single developmental domain, one mother spent less than 4 h per day caring for her child, and 2 mothers showed potential rating biases, with one mother giving high ratings for all DMQ items and one mother rating all items with very low scores. Thus, 52 dyads with and without GDD (each group was 26) were considered to have valid data for analyses in this study.

### 2.3. Measures

#### 2.3.1. Young Children’s Participation and Environment Measure (YC-PEM)

The YC-PEM is a questionnaire comprising 28 items designed to measure the participation of young children in a variety of activities within the home (13 items), daycare/preschool (3 items), and the community (12 items) [13]. It is rated by caregivers of children aged 0–5 years [8]. For each participation item, parents indicate the frequency of their child’s engagement in the activity (ranging from 0 = never to 7 = once or more daily), the child’s level of involvement during participation (ranging from 1 = minimally involved to 5 = highly involved), and whether they wish to see a change in their child’s participation (yes or no; if yes, parents can specify the type(s) of desired change) [7,13,14]. The YC-PEM demonstrated adequate psychometric characteristics, including acceptable test-retest reliability (ICC = 0.65–0.90) and construct validity, as referenced in studies [7,13,14]. Furthermore, it has undergone cultural adaptation to suit parents in Chinese-speaking countries, as noted in references [7,35].

In this study, two types of summary scores from the participation scales (frequency and involvement) can be calculated by each of the three settings (home, daycare, and community) in the YC-PEM. The score calculation is detailed in Khetani et al. study [8]. The higher score indicated higher participation attendance and participation involvement.

#### 2.3.2. The Revised Dimensions of Mastery Questionnaire (DMQ 18)

The DMQ 18 has been commonly used to measure mastery motivation in many countries around the world, and it assesses the motivation of young children using ratings by an adult familiar with the child (parent, caregiver, or teacher) [24]. Six scales (cognitive persistence, gross motor persistence, social persistence with adults, and social persistence with children; mastery pleasure and negative reactions to challenge) are used to assess mastery motivation. Every question in the DMQ 18 is assessed using a five-point Likert scale, ranging from 1, indicating “not at all like this child”, to 5, signifying “exactly like this child”. In addition to the six main scales for assessing mastery motivation, a total persistence score can be computed based on the average of four persistence scales; the total social persistence score is derived from the average of the two social persistence scale scores. Higher scores indicate higher mastery motivation [24]. The reliabilities of the four persistence scales, along with the mastery pleasure scale and the negative reaction to challenge scale, ranged from marginally acceptable to good, including adequate internal consistency (Cronbach’s α ≥ 0.70) and test-retest reliability (r = 0.73–0.89) [25]. Good convergent validity and construct validity of the DMQ 18 have been reported in several studies [36,37,38]. In this study, we used total persistence, mastery pleasure, and negative reaction to challenge as indicators of mastery motivation.

#### 2.3.3. Behavior Rating Inventory of Executive Function—Preschool (BRIEF-P)

The BRIEF-P is widely used to assess executive function skills of young children, which is completed by one caregiver. The BRIEF-P is a questionnaire comprising 63 items designed to evaluate child performance related to executive function in daily activities within natural settings for preschool-aged children with an age range of 2–5 years [39]. Caregivers characterize their child’s behavior using a 3-point Likert scale, denoting the frequency with which the child exhibited certain behavior (1 = never, 2 = sometimes, 3 = often). There are five clinical scales: inhibit, shift, emotional control, working memory, and plan/organization. The Global Executive Composite (GEC) is a summary score. The GEC and the scores for all five clinical scales are transformed into standardized T scores, with a mean of 50 and a standard deviation of 10. The BRIEF-P has shown adequate internal consistency reliability (Cronbach’s alpha = 0.80–0.95) and test–retest reliability (*r* = 0.78–0.90) [40]. It also has shown acceptable convergent and discriminant validity with measures of hyperactivity/impulsivity, somatic complaints, inattention, depression, and anxiety [39,40]. In this study, the GEC scores of the BRIEF-P were used to represent executive function skills.

#### 2.3.4. Comprehensive Developmental Inventory for Infants and Toddlers (CDIIT)

The CDIIT is a standardized diagnostic developmental test with a normative sample consisting of 3703 Taiwanese children aged 3 to 72 months. It comprises six developmental subtests, namely cognition, language, gross motor, fine motor, social, and self-help. The cognitive subtest assesses a child’s cognitive abilities; the language subtest encompasses both comprehension and expression aspects. The gross motor subtest evaluates anti-gravity movements, locomotion, and coordination of body movements, whereas the fine motor subtest covers items related to fundamental hand dexterity and visual-motor coordination. The social subtest assesses social interaction, and the self-help subtest includes items related to skills like feeding, dressing, and hygiene [41]. The examiner administers all the cognitive and motor-related items, as well as select items from the language subtests. The main caregiver rates the remaining items from the language subtest, as well as the social and self-help subtests.

Each test item is scored as either 0 or 1, with a score of 1 indicating success either during the test or based on the caregiver’s observation. Developmental ages and developmental quotients for all subtests, as well as the entire test, were determined according to Taiwanese norms [41]. A developmental quotient (DQ) less than 70 (2 SD below the mean) on a subtest indicates developmental delay in this study. For children developing typically, their whole DQ from the CDIIT above 85 indicates typical development. It takes about 40 min to administer CDIIT. The CDIIT has adequate psychometric properties, including test-retest reliability (ICC 0.89–0.99), construct validity [42], and concurrent validity [43]. In this study, the whole DQ was used to assess child developmental abilities.

### 2.4. Study Procedure

This study received approval from the Human Subjects Review Committee at the participating medical center (IRB/REC code: CMUH109-REC1-032). Mothers and children were invited to the laboratory for a 90-min session. Following a warm-up period, a trained pediatric physical therapist conducted the CDIIT, with a 5-min break afterward. The mental age of each child was promptly calculated post-test and utilized to determine an initially appropriate difficulty level for subsequent mastery tasks. Following another 5-min break, the child underwent testing using the individualized structured mastery task method (data from which would be presented in another study). Concurrently, the mother, positioned with her back facing the child in the same room, provided basic demographic information (such as maternal education, family income, and socioeconomic status determined by the father’s occupation and education). Additionally, the mother completed three questionnaires (YC-PEM, DMQ 18, BRIEF-P).

### 2.5. Data Reduction and Analysis

All outcome variables, including mastery motivation, executive function variables, and Developmental Quotients (DQs), were examined for normality and were subjected to statistical analysis using IBM SPSS software (version 25). If continuous variables were normally distributed, relevant parametric analyses would be used. If continuous variables were not normally distributed, relative nonparametric tests would be used [44]. Descriptive statistics were utilized to provide fundamental information regarding the children, their families, and the scores obtained from various assessments (see Table 1). The main dependent variables were six participation indicators: participation frequency and participation involvement at each of the three settings (home, daycare, and community). The total persistence, mastery pleasure, and negative reaction to challenges scores were measured by mothers’ ratings on the DMQ 18 to obtain mastery motivation variables. The overall executive function skill scores were used to indicate the executive function variable.

Regarding family demographics, mothers reported their own education levels using a 7-point scale, with 1 indicating illiteracy; 2, primary school; 3, junior high school; 4, senior high school; 5, college; 6, bachelor’s degree; and 7, postgraduate degree. These 7 levels comprised the maternal education score. Family income was coded as 2 levels, with 1 indicating <100,000 NTD/year; 2, ≥100,000 NTD/year.

To compare the differences between the two groups, independent *t*-tests were used for continuous variables with a normal distribution. Indeed, for independent *t*-tests, effect sizes (ES) were determined using the formula (*d* = mean difference in two paired groups/standard deviation of the paired differences) [44]. One-way ANOVA and paired *t*-tests were used to examine the participation differences among three settings (home, preschool, and community) in young children with GDD. Pearson correlations were used to analyze the correlations of children’s mastery motivation and executive function with children’s frequency and involvement of participation in three settings. To further investigate the disparities between the groups, Fisher’s r-to-z transformation analysis was employed to determine the significance of the distinction between correlation coefficients in the GDD and TD groups.

The correlations of participation with mastery motivation and executive function in GDD and TD groups were analyzed using a Pearson correlation. Hierarchical regression models were conducted to examine the significance of predictors of 6 outcome variables (participation frequency and involvement in three settings) for the whole sample, respectively, after considering the contribution of other predictors. Child health conditions indicated group differences (GDD, TD). Child total persistence scores, mastery pleasure, and negative reaction to challenges were indicated as mastery motivation. Child total executive function scores were presented as the child’s overall executive function skills. Variables that demonstrated significance in the bivariate correlation tests (significance level: α < 0.05, two-tailed) were included as independent variables in the regression model. If there were several models, the final model was determined by the significant β and F values and significant F change, which is used to tell us whether these additional variables significantly improved on the previous model.

## 3. Results

### 3.1. Group Characteristics

Table 1 presents the descriptive statistics for children diagnosed with GDD aged 24–55 months and typically developing (TD) children between 15–29 months, including details regarding their families. Besides GDD, 13 children had other medical diagnoses, including Down syndrome (*n* = 6), Williams syndrome (*n* = 1), microcephalus (*n* = 1), and genetic disorders (*n* = 5). Even though there were notable differences in the developmental quotients (DQs) between the two groups, developmental age on various domains of the CDIIT did not differ, except in the language and social domain (refer to Table 1). According to the norms in the CDIIT manual for the GDD group, four children were classified in the borderline range for motor delay, six for cognitive delay, and two for language delay. In the mild range, there were ten with motor delay, six with cognitive delay, and thirteen with language delay. In the moderate range, there were twelve with motor delay, sixteen with cognitive delay, and two with language delay.

There were no significant differences in family variables between the GDD and TD groups, including mothers ‘age, socioeconomic status, yearly family income, and maternal education. The majority of mothers in both groups (*n* = 34, 69%) had obtained a college or graduate degree. The classification of family socioeconomic status (SES) was established according to the father’s educational attainment and occupational standing, ranging from I to IV, with I denoting the highest SES [45]. The majority of families in this study belonged to the middle to high socioeconomic class.

### 3.2. Group Comparisons of Child Daily Participation

The results of comparisons between the global delay and typical groups on the YC-PEM scales are presented in Table 2. Young children with global delays showed significantly lower participated attendance in daycare centers than children developing typically (*t* = 3.25, *p* < 0.05, using independent *t*-tests). There were no group differences in participated attendance at home and in community settings. For participated involvement, young children with global delays were rated significantly lower involvement at home, daycare centers, and community settings than children with typical development.

For exploring participation differences among three settings (home, preschool, community) in young children with GDD, we found the highest participation frequency at home settings than the other two settings (*t* = 2.01 to 11.39, *p* < 0.05, using paired *t*-tests). Young children with GDD were found to show a lower level of involvement in daycare settings when compared to community settings (*t* = 2.44, *p* < 0.05, using paired *t*-test). There were no significant differences between the home setting and the daycare setting/community setting.

### 3.3. Correlations of Mastery Motivation and Child Daily Participation

The results revealed that high persistence scores were significantly related to participation frequency at home and daycare settings in the whole group (*r* = 0.30 to 0.33). We also found that high persistence scores and mastery pleasure were positively related to participation involvement at home and community settings in the whole group (*r* = 0.43 to 0.59). Higher negative reactions to challenges were significantly related to participation frequency and involvement at home and community settings in the whole group (*r* = 0.33 to 0.56). However, the correlations between child mastery motivation indicators and child participation indicators were different in the two groups. In the GDD group, high persistence and pleasure were positively related to participation frequency and involvement at home and in community settings. Within the Typically Developing (TD) group, significant correlations were observed between negative reactions to challenges and participation involvement in daily activities at home, daycare, and community settings (Table 3). Regarding examining the correlation coefficients across the two groups, the findings revealed no significant differences in the magnitudes of the correlation coefficients (z = −1.25 to 1.25, *p* = 0.21 to 0.34). Consequently, it appears that the association between mastery motivation and daily participation is relatively similar in both groups.

### 3.4. Correlation between Executive Function and Child Daily Participation

In the whole sample, high overall executive function scores were negatively associated with participation involvement at home, daycare, and community settings (*r* = −0.34 to −0.54) but not participation frequency in the three settings. Specifically, for the TD group, high overall executive function scores were associated with low participation frequency scores at home and in community settings (*r* = −0.39 to −0.41). However, no significant correlations between overall executive function and participation indicators were found in the GDD group. According to the Fisher r-to-z transformation analysis, there were no differences in the values of the correlation coefficients between the GDD and TD groups (z = −1.62–0.90, *p* = 0.11–0.88) were found. These results indicate that the negative correlations between executive function scores and participation scores in the three settings were similar in young children with GDD and TD.

### 3.5. Possible Child Predictors for Child Daily Participation

Regarding the contribution of child health condition, developmental abilities, mastery motivation, and executive function to child daily participation, we further used hierarchical regression to examine the possible contribution of the above predictors on child daily participation at home, daycare, and community settings. There were no significant predictors of child daily participation frequency in the home setting. We found that child mastery pleasure (*β* = 0.38, *p* < 0.05) was a significant predictor of child daily participation involvement in the home setting and explained 51% of the variance for participation involvement scores. It indicated that young children with and without GDD who experienced higher mastery pleasure showed higher levels of involvement when participating in daily activities in the home setting.

For the daycare setting, the child’s health condition significantly predicted participation frequency scores on the YC-PEM (*β* = −0.71, *p* < 0.05). We found that the child’s health condition (*β* = −0.63, *p* < 0.05) and child’s overall persistence (*β* = 0.29, *p* < 0.05) were significant predictors of participation involvement in the daycare setting, and they explained 34% of the variance for participation involvement scores in the daycare setting. It indicated that young children with mild delay and higher persistence showed a higher level of involvement in the daycare setting. There were no significant predictors for the participation frequency and involvement in the community setting. Thus, somewhat different predictors of participation frequency and involvement at home, daycare, and community settings were shown in this study.

## 4. Discussion

The main findings of this study indicated that young children with GDD exhibited significantly lower frequency of participation at daycare centers compared to mental age- and sex-matched children in the TD group. However, no significant group differences were observed in the home or community settings. Moreover, when compared to a mental age- and sex-matched TD group, young children with GDD showed significantly lower participation involvement at home, daycare, and community settings, especially the largest group differences in participation involvement at home. For describing participation patterns in young children with GDD, young children with GDD showed the highest participation frequency at home than in daycare and community settings. Young children with GDD showed the lowest participation involvement in daycare settings when compared to home or community settings.

For the association between child mastery motivation, executive function, and participation level in young children, child mastery pleasure significantly predicted participation involvement in home settings. Child health condition was a significant predictor of participation frequency and involvement in daycare settings. Child persistence significantly predicted participation involvement in daycare settings. The following section explores potential reasons for the main findings and discusses clinical implications.

One of the key findings was that young children with GDD showed lower participation frequency at daycare centers than those of the mental age-matched TD group, but no group differences in home or community settings. Lower participation involvement at home than in daycare and community settings was found in the GDD group when compared to the TD group. Thus, our hypothesis about group differences in participation components was supported. Our finding is consistent with and extends previous studies about the effect of child health conditions on children’s involvement in daily activities [6,13,14]. The possible reason was that young children with delays were reported to have behavioral problems. Previous studies have shown that young children’s behavior was positively associated with their adaptation in out-of-home contexts. Young children’s participation levels depend on their parents. Then, parents frequently reported that children’s behavioral challenges affected their child’s participation in daycare or community settings because they consumed energy to manage their child’s problematic behaviors in uncertain situations [8,20,46]. Another reason was that parents of children with disabilities reported that they met more barriers to participation and experienced lower social support, education quality, activity intensity, and activity enjoyment [6,14,47,48]. When exploring the participation level of young children with GDD, the highest participation frequency and involvement were reported for the activities in a home setting. Home is the most prominent environmental contest [39]. Parents of children with disabilities may have different expectations about participation involvement at daycare or community settings for their children at a young age. Thus, it is why families of children with global delays have seldom participated in various activities outside of the home setting.

Another key finding in this study was that young children who experienced higher mastery pleasure showed higher participation involvement at home. This finding is consistent with some previous studies in children with delays [19,28]. The above finding has indicated that a child with high positive affect might prefer to do various challenging tasks, such as moving over to obtain an object. Furthermore, delay with lower severity levels means that a child could have sufficient developmental abilities to achieve a goal. We also found that in a daycare setting, a child’s health condition is a significant predictor of participation frequency and involvement. The possible reason was that parents of children with more severe levels of developmental delay might not be able to do well in daily routines, such as eating, toileting, and clothing [9,15,19,26,27]. What’s worse, children with more severe levels of delays usually exhibited problematic behaviors in daily activities and required assistive technology to engage in outdoor activities. Therefore, parents usually hesitate to take their kids to daycare centers to avoid being attended by teachers or spending too much time using equipment for outdoor activities. In daycare settings, children with higher persistence showed better participation involvement. In a daycare setting, teachers usually provide children with tasks that could be enjoyable and interesting to them. While the community environment may present greater challenges, further study might be required for possible environmental factors influencing child participation level in addition to child factors.

In this study, we found that there was no significant association between overall executive function skills and daily participation in young children. It is possible that the daily activities of young children are organized and regulated by their primary caregivers, leading to a situation where the executive function has a less direct impact on the daily participation of these children. Executive function may play a more significant role in the participation of older children, who are anticipated to be more self-reliant [49]. While overall executive function skills were positively associated with participation frequency at home and in community settings in young children with TD, it indicated that young children developing typically showed better high-level cognitive processes required for complex goal-directed behavior to participate in daily activities at home or in the community [30,31,32,34].

Regarding the clinical implication, children’s mastery motivation and health condition showed positive predictive effects on daily participation in three settings. It indicated that young children with difficulties in performing tasks may also show low mastery motivation during the process of participating in daily routines. Thus, it is crucial for early interventionists/educators to coach parents on how to observe and facilitate their child’s motivation based on solution-focused therapy and a strength-based thinking approach. Presently, the Kids’ Skills program has been devised to aid children in learning responsibility and recognizing their individual strengths to enhance their motivation for engaging in daily activities [50,51]. The Kids’ Skills program encompasses fifteen steps, progressing through phases such as skill training (steps 1–2), motivational phases (steps 3–10), skill practice (steps 11–13), and reinforcement of skill learning (steps 14–15). The specific descriptions of each step were demonstrated in Furman’s study [51]. In this study, we focused on the motivational phase to encourage the daily participation of young children. The core concept of the motivation phase was to make the child have a sense of autonomy to choose the skill. Then, they would be guided by adults to learn skills through emphasizing their strengths and massive practice. Therefore, the child would have the belief that they should be capable of doing skilled activities. Another is that early interventionists/educators should pay attention to the child’s health condition because health conditions may influence parents’ expectations about their child’s participation levels and possible surrounding barriers influencing participation levels of young children [7,9,15,24].

There were several limitations as follows: (1) our study design was a case-control study, and relative lack of longitudinal research on the participation patterns of young children with disabilities; (2) we only used parental reports to measure child mastery motivation and executive function as well as participation in daily activities, and it is necessary to include both behavioral tasks and parental report to fully understand the possible mechanism; (3) due to small sample size it was not possible to make more advanced analyses, and results might only generalize to middle to upper SES Asian populations because of sample homogeneity [22].

## 5. Conclusions

In this study, young children with GDD showed significantly lower participation involvement at home, daycare, and community settings, especially the largest group differences in participation involvement at home when compared to a mental age- and sex-matched TD group. Furthermore, young children with global delays showed significantly lower participation frequency at the daycare center than in the other two settings. Another finding was that high child mastery pleasure and better child health conditions were significantly associated with better participation frequency and involvement at home and daycare settings in young children with and without GDD. Therefore, it is potentially crucial for educators and clinicians to guide parents not only in attending to their child’s developmental status but also in coaching them on how to enhance their child’s mastery motivation. This approach aims to foster young children’s active engagement in daily activities across various settings.

## Figures and Tables

**Table 1:** Characteristics of the children with global developmental delays and with typical development (*n* = 26 for each group).

Variables	GDDs	TD	*t* ^a^	*p*	*d*
**Child variables**					
Age (months) ^a^	41.2 (9.4)	32.2 (10.4)	3.28	<0.001	0.91
Male gender (*n*, %)	14 (64%)	14 (64%)			-
DQ of CDIIT ^a^					
Whole DQ	59.8 (8.2)	99.2 (10.9)	14.67	<0.001	4.08
Cognitive DQ	61.3 (9.2)	94.4 (10.1)	12.39	<0.001	3.43
Motor DQ	59.4 (8.9)	88.8 (17.1)	7.79	<0.001	2.16
DA of CDIIT (months) ^a^					
Cognitive DA	26.0 (7.6)	30.1 (9.8)	1.69	0.10	0.47
Language DA	25.9 (7.5)	33.7 (10.9)	3.02	<0.001	0.83
Gross motor DA	25.0 (7.9)	29.3 (11.7)	1.55	0.13	0.43
Fine motor DA	27.4 (8.1)	30.1 (11.4)	1.01	0.32	0.27
Social DA	23.4 (8.7)	41.1 (14.7)	5.29	<0.001	1.47
Self-care DA	29.2 (12.2)	35.0 (14.8)	1.54	0.13	0.43
BRIEF-P GEC scores	67.2 (10.8)	51.7 (7.7)	5.48	<0.001	1.65
DMQ 18 Total Persistence	3.1 (0.74)	3.7 (0.56)	3.46	<0.001	0.91
DMQ 18 Mastery Pleasure	4.1(0.81)	4.6 (0.39)	2.42	0.02	0.79
DMQ 18 Negative Reaction to Challenges	3.5 (0.85)	3.5 (0.58)	0.19	0.85	0.00
**Family variables**					
Caregivers’ age (years) ^a^	35.6 (5.2)	34.0 (4.1)	1.19	0.12	0.34
Caregivers’ education level (*n*, %)					
≥college	14, 54%	22, 85%	-	0.06	-
Socioeconomic status (Class I and II; *n*, %)	12,	19,	-	0.18	-
Annual income (≥1,000,000 NTD; *n*, %)	17,	9,	-	0.13	-

Note: ^a^ an independent *t*-test (two-tailed); All measurements are expressed as mean (SD); *d* = mean difference in two groups/standard deviation of the differences. Abbreviations: BRIEF-P = Behavior Rating Inventory of Executive Function—Preschool; CDIIT = Comprehensive Developmental Inventory for Infants and Toddlers; DA = developmental age; DQ = developmental quotient; DMQ 18 = Revised Dimensions of Mastery Questionnaire; GDD = Global Developmental Delays; TD = Typical Development.

**Table 2:** Comparison of mental age-matched children with and without global developmental delay (GDD) on the participation in different settings.

YC-PEM Scales	GDD	TD	*t* ^a^	*p*	*d*
(*n* = 26)	*M* (*SD*)	*M* (*SD*)			
Home frequency	4.75 (1.16)	5.21 (0.91)	−1.60	0.12	0.44
Home involvement	3.01 (0.76)	4.00 (0.76)	−4.67	<0.001	1.30
Daycare center frequency	3.64 (2.30)	5.51 (1.50)	−3.25	<0.01	0.96
Daycare center involvement	2.38 (1.62)	4.33 (1.20)	−04.74	<0.001	1.37
Community frequency	2.74 (0.69)	2.65 (1.01)	0.38	0.71	0.10
Community invovlement	3.06 (0.98)	3.70 (1.12)	2.22	0.03	0.61

Note: ^a^ an independent *t*-test (two-tailed); All measurements are expressed as mean (SD); *d* = mean difference in two groups/standard deviation of the differences.

**Table 3:** Relationship between mastery motivation, executive function, developmental ability and daily participation in young children with and without global developmental delay.

		Home				Daycare Center				Community			
		Frequency		Involvement		Frequency		Involvement		Frequency		Involvement	
Variables	Indicators	GD	TD	GD	TD	GD	TD	GD	TD	GD	TD	GD	TD
Mastery Motivation	Total *p*	0.60 **	0.28	0.59 **	0.30	0.17	0.15	0.25	0.35	0.39 *	0.13	0.39 *	0.54 **
	Pleasure	0.60 **	−0.05	0.67 **	0.34	0.36	−0.28	0.23	−0.09	0.60 **	0.00	0.57 **	0.29
	Neg Rec.	0.43 *	0.15	0.42 *	0.39 *	0.10	0.15	0.18	0.50 **	0.58 **	0.42 **	0.56 **	0.42 **
EF	Overall EF	−0.17	−0.41 *	−0.28	−0.37	−0.32	−0.28	−0.12	−0.31	−0.12	−0.39 *	−0.09	−0.37
Development ability	Total DQ	0.19	0.00	0.04	0.19	−0.20	−0.02	0.00	0.09	0.36	0.09	0.06	0.27

Note: each GDD and TD group = 26; Correlations analyzed by Pearson Correlations; * *p* < 0.05; ** *p* < 0.01. Abbreviations: EF = executive function; GD = global developmental delay; *p* = persistence; Pleasure = Mastery Pleasure Scale scores from the DMQ 18; Neg Rec. = Negative Reaction to Challenges; TD = typical development.

## Data Availability

Data are not publicly available due to privacy and ethical restrictions.
